# Evaluation of the quality of multiple-choice questions according to the students’ academic level

**DOI:** 10.1186/s12909-022-03844-3

**Published:** 2022-11-11

**Authors:** Mercedes Iñarrairaegui, Nerea Fernández-Ros, Felipe Lucena, Manuel F. Landecho, Nicolás García, Jorge Quiroga, Jose Ignacio Herrero

**Affiliations:** 1 Liver Unit, Clínica Universidad de Navarra, Av. Pio XII, 36, 31008 Pamplona, Spain; 2 Instituto de Investigación Sanitaria de Navarra (IdiSNA), Pamplona, Spain; 3 Centro de Investigación Sanitaria en Red de Enfermedades Hepáticas y Digestivas, Madrid, Spain; 4 Department of Internal Medicine, Clínica Universidad de Navarra, Pamplona, Spain

**Keywords:** Academic level, Assessment, Difficulty, Discrimination, Multiple choice question, Pathophysiology

## Abstract

**Background:**

One of the most important challenges in medical education is the preparation of multiple-choice questions able to discriminate between students with different academic level. Average questions may be very easy for students with good performance, reducing their discriminant power in this group of students. The aim of this study was to analyze if the discriminative power of multiple-choice questions is different according to the students’ academic performance.

**Methods:**

We retrospectively analyzed the difficulty and discrimination indices of 257 multiple-choice questions used for the end of course examination of pathophysiology and analyzed whether the discrimination indices were lower in students with good academic performance (group 1) than in students with moderate/poor academic performance (group 2). We also evaluated whether case-based questions maintained their discriminant power better than factual questions in both groups of students or not. Comparison of the difficulty and discrimination indices between both groups was based on the Wilcoxon test.

**Results:**

Difficulty index was significantly higher in group 1 (median: 0.78 versus 0.56; *P* <  0.001) and discrimination index was significantly higher in group 2 (median: 0.21 versus 0.28; *P* <  0.001). Factual questions had higher discriminative indices in group 2 than in group 1 (median: 0.28 versus 0.20; *P* <  0.001), but discriminative indices of case-based questions did not differ significantly between groups (median: 0.30 versus 0.24; *P* = 0.296).

**Conclusions:**

Multiple-choice question exams have lower discriminative power in the group of students with high scores. The use of clinical vignettes may allow to maintain the discriminative power of multiple-choice questions.

## Background

Assessment is an essential part in medical education. It is important, not only as a mean of scoring, but also as a feedback mechanism. One of the most used methods of assessment is a written exam based, at least in part, on multiple choice questions (MCQ). A MCQ exam is objective and allows asking questions about a wide range of areas. Furthermore, it is easy to correct in large groups of students.

One of the most important challenges in the preparation of a MCQ is to construct a question with adequate difficulty level and the ability to discriminate between performers and non-performers [1]. Discrimination is not only essential for identifying those students who are competent or not. Discrimination among those who pass an exam is also important because their scores may give them better opportunities of choosing better residency programs. These aims are obtained more frequently by reducing the number of options [2], in order to decrease the number of non-functioning distractors [3]. For large groups, discrimination and difficulty indices are assumed to be stable [4, 5], but this is not the case when the same exam is administered to small cohorts [6]. It is likely that discrimination indices are different between performers and non-performers, because low-scoring students choose distractors more frequently than high-scorers [7]. MCQ are usually used only as context-free questions [8] aimed at repetition of factual material [9], but MCQ that use clinical vignettes may also allow to explore higher cognitive levels [10, 11].

The aim of this study was to analyze if the discriminative power of MCQ is different in high-scoring students than in low-scoring ones. We also analyzed whether MCQ based on clinical vignettes, that explore higher cognitive levels, were more discriminative than factual questions.

## Methods

### Study setting

The curriculum of Medicine at the Universidad de Navarra is distributed over 6 years. Until 2020, it was divided in three pre-clinical years and three clinical years. Pathophysiology is included in the third year, and it is conceived as the cornerstone of the transition between pre-clinical and clinical years. Professors of this one-year course are members of the Department of Internal Medicine. The course has around 200 students every year, and it is divided in two periods (September to December, and January to May). The first semester includes blood, kidney, cardiovascular and respiratory pathophysiology, and the second one includes gastrointestinal, hepatic, neurological, endocrinological and metabolic pathophysiology.

The assessment of the course is done with two mixed written exams that include a MCQ test and the discussion of a clinical case. They represent 80 and 20%, respectively, of the exam score. All the MCQ have four potential answers, and only a right one. Correct answers score one point, whereas the failure in a question subtracts 1/3. At the end of the first semester, the students have an exam of the contents that have been given until then. Those students who achieve a score above 6 out of 10 in this exam earn the right to take a final exam with a MCQ test that includes only questions about the contents covered in the second semester, while the rest of the students will face a MCQ test that include the whole contents of the course. So, the final exam includes the discussion of a clinical case, that is common for all the students (20% of the score) and a MCQ test that is different for those students who achieved a score above 6/10 than the MCQ test of the rest of the class. The first ones have a test of 75 MCQ that includes only the contents covered in the second semester, and the second ones have a test with 100 MCQ that includes all the course. Thus, approximately 50 MCQ are common in both tests. The duration of the exam is adjusted to the number of questions.

### Study design

This retrospective study compares the difficulty and discrimination indices of the MCQ that were common in both final exams in the years 2015–16, 2016–17, 2017–18, 2018–19, and 2020–21. The exam of the year 2019–20 was excluded because it was done in remote, because of the COVID19 pandemic restrictions. Two groups were compared: group 1 included the students that had passed the first semester exam with a score above 6/10, and group 2 included the students who scored less than 6/10 in the first semester exam. The number of students in group 1 in each of the years studied were: 28 in 2016, 36 in 2017, 34 in 2018, 40 in 2019, and 36 in 2021 (*N* = 146). Group 2 included 74 students in 2016, 86 in 2017, 86 in 2018, 68 in 2019, and 72 in 2021 (*N* = 314). These numbers include only the students that were within the 27% of the students with the higher scores and the 27% with lower scores of each group. The proportion of students that were included in group 1 each year were 27% in 2016, 29% in 2017, 28% in 2018, 37% in 2019, and 33% in 2021.

We obtained the difficulty and the discrimination indices for each question, evaluating the 27% of the students with the higher scores and the 27% with lower scores in each group. Difficulty index was defined as the relative frequency of the students who chose the correct response (i.e. a difficulty index of 0.4 indicates than the question was answered correctly by 40% of the students). Discrimination index was defined as the difference in correct answers for a given question between the 27% higher-scorers and the 27% lower-scorers divided by the number of students in each of these subgroups [12]. Distractors that were chosen by less than 5% of the students were considered poor functioning, and those that were not chosen by any student were considered non-functioning [13]. We also analyzed whether questions based on clinical scenarios -as compared to factual questions-, and whether easy questions, defined as those with a difficulty index above 0.7 [14] -as compared with non-easy questions- had a different discrimination index in both groups.

### Statistical analysis

Continuous variables are expressed as median (quartile range) and categorical variables as number (percentage). Comparison of the difficulty and discrimination indices between both groups was based on the Wilcoxon test. Comparison of the proportions of poor-functioning and non-functioning distractors between both groups was done with the chi-square test. A *P* value below 0.05 was considered statistically significant. All the data were analyzed with the software SPSS Statistics for Windows version 20.0 (IBM Corp., Armonk, NY).

Ethical approval was obtained by the Universidad de Navarra Ethics Committee for Research (project 2021.134). Data were obtained and recorded in an anonymous database, without any personal information.

## Results

The number of MCQ analyzed was 257. Factual questions were more frequent than those based on clinical scenarios: 198 (77%) versus 59 (23%).

### Analysis of difficulty index

**Fig. 1:**
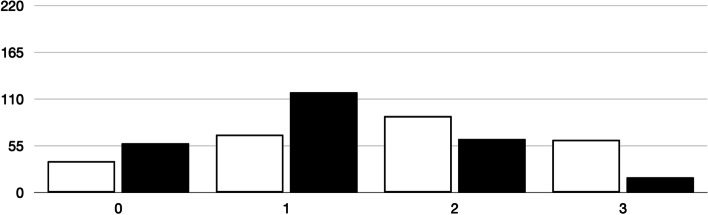
Number of non-functioning distractors in 257 MCQ (three distractors per question) in the final exam, according to the students’ results in the first semester exam* (comparison between groups: *P* < 0.001). *Group 1 (white bars). Students with a score above 6/10 in the first semester exam. Group 2 (black bars). Students with a score below 6/10 in the first semester exam

**Fig. 2:**
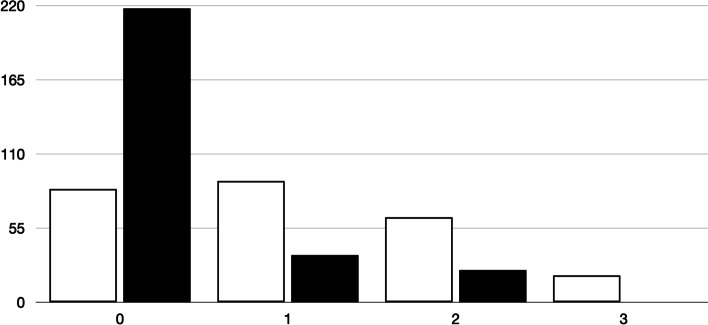
Number of poor functioning distractors in 257 MCQ (three distractors per question) in the final exam, according to the students’ results in the first semester exam* (comparison between groups: *P* < 0.001). *Group 1 (white bars). Students with a score above 6/10 in the first semester exam. Group 2 (black bars). Students with a score below 6/10 in the first semester exam

Difficulty indices are shown in Table 1. As expected, these indices were higher for group 1 than for group 2, and this global significant difference was maintained for factual and case-based questions. The differences between group 1 and 2 were statistically significant every year of the study (0.79 versus 0.54 in 2016; *P* <  0.001; 0.78 versus 0.52 in 2017; *P* <  0.001; 0.75 versus 0.49 in 2018; *P* <  0.001; 0.73 versus 0.57 in 2019; *P* <  0.001; and 0.83 versus 0.60 in 2021; *P* <  0.001). However, intragroup differences in the difficulty indices between factual and case-based questions were not significantly different. Fifty-two (20%) of the questions were considered easy, as their difficulty index was 0.7 or higher. Ten case-based questions (17%) and 42 factual questions (22%) were easy (*P* = 0.474). The number of poor functioning and non-functioning distractors were higher in group 1 than in group 2 (Figs. 1 and 2).

**Table 1:** Comparison in the difficulty indices of 257 MCQ in the final exam of pathophysiology between students with high scores (group 1) and low scores (group 2)

	Group 1^a^	Group 2^b^	*P*
All the questions (*N* = 257)	0.78 (0.62–0.89)	0.56 (0.37–0.67)	< 0.001
Factual questions (*N* = 198)	0.79 (0.65–0.89)	0.56 (0.41–0.67)	< 0.001
Case-based questions (*N* = 59)	0.75 (0.56–0.89)	0.49 (0.32–0.67)	< 0.001
P***	0.125	0.102	

***Significance (factual versus case-based questions)

^a^Group 1. Students with a score above 6/10 in the first semester exam

^b^Group 2. Students with a score below 6/10 in the first semester exam

### Analysis of discrimination index (Table 2)

Discrimination indices were significantly higher in group 2 than in group 1. The median value was lower for group 1 than for group 2 every year of the study (0.21 versus 0.32 in 2016; *P* = 0.017; 0.22 versus 0.23 in 2017; *P* < 0.618; 0.18 versus 0.27 in 2018; *P* = 0.158; 0.21 versus 0.25 in 2019; *P* = 0.331; and 0.17 versus 0.33 in 2021; *P* = 0.003). This difference was especially evident when the discriminative indices of either factual or easy questions were compared between groups. Easy questions were significantly less discriminative than non-easy questions in group 1, but not in group 2. When non-easy questions were specifically analyzed, the differences in the discriminative indices of factual questions between group 1 and group 2 were close to significance. These differences were not found when the discriminative indices of case-based questions were compared between groups.

**Table 2:** Comparison in the discrimination indices of 257 MCQ in the final exam of pathophysiology between students with high scores (group 1) and low scores (group 2)

	Group 1^a^	Group 2^b^	*P*
All questions (*N* = 257)	0.21 (0.11–0.34)	0.28 (0.18–0.37)	< 0.001
Factual (*N* = 198)	0.20 (0.11–0.33)	0.28 (0.18–0.37)	< 0.001
Case-based (*N* = 59)	0.24 (0.11–0.35)	0.30 (0.19–0.37)	0.296
P***	0.300	0.620	
Easy (*N* = 52)	0.11 (0.06–0.17)	0.28 (0.19–0.36)	< 0.001
Non-easy (*N* = 205)	0.24 (0.14–0.36)	0.28 (0.18–0.38)	0.117
P****	< 0.001	0.663	
Non-easy, factual (*N* = 156)	0.22 (0.14–0.36)	0.28 (0.18–0.38)	0.071
Non-easy, case-based (*N* = 49)	0.28 (0.16–0.39)	0.28 (0.18–0.37)	0.972
P*****	0.481	0.957	

***Significance (factual versus case-based questions)

****Significance (easy versus non-easy questions). Easy questions: difficulty index equal or higher than 0.7 in both groups

*****Significance (non-easy factual versus case-based questions)

^a^Group 1. Students with a score above 6/10 in the first semester exam

^b^Group 2. Students with a score below 6/10 in the first semester exam

**Fig. 3:**
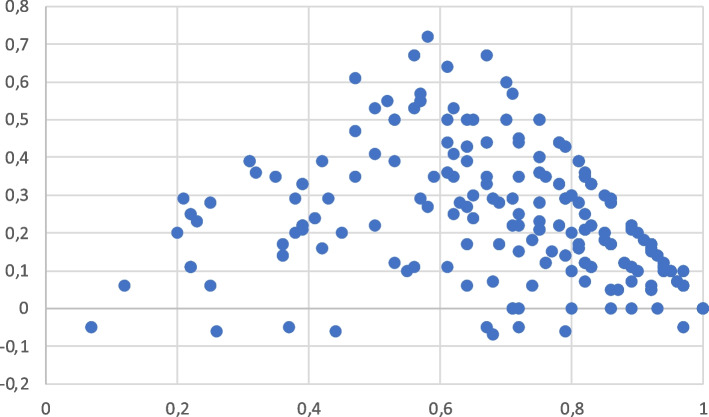
Plotting of discriminative indices (X axis) versus difficulty indices (Y axis) in group 1

**Fig. 4:**
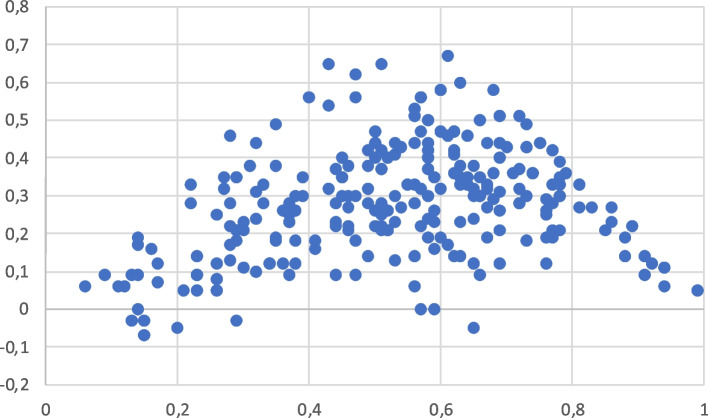
Plotting of discriminative indices (X axis) versus difficulty indices (Y axis) in group 2

Plotting discrimination against difficulty (Figs. 3 and 4) shows that, for both groups, questions with difficulty indices between 0.4 and 0.7 had the highest discriminative indices, and questions with difficulty indices higher than 0.7, and lower than 0.4 had a progressive reduction of their discriminative power. The proportion of questions that should be eliminated or completely revised, according to a discrimination index lower than 0.2 [12] in group 1 was 39.3% for those with a difficulty index up to 0.4, 18.7% for questions with a difficulty index between 0.41 and 0.7, and 57.6% for questions with a difficulty index above 0.7. In group 2, the proportion of questions with a discrimination index lower than 0.2 was 53.5% for questions with a difficulty index up to 0.4, 16.1% for questions with a difficulty index between 0.41, and 0.7, and 27.5% for questions with a difficulty index higher than 0.7.

## Discussion

The most relevant result of this study is the finding that MCQ have lower discrimination capacity in students with high scores. This finding opposes to the classical thought that suggests that the discrimination and the difficulty of a question are stable [15, 16]. Young et al. found that discrimination indices for items are variable when they are administered to small cohorts [6]. Our study also suggests that the discrimination indices are also dependent on the academic level of the students. The discrimination index of a MCQ is lower in students with high scores because they choose distractors less frequently than the students with low scores [7]. This finding is also in agreement with a recent paper that shows that the reliability of easy exams is lower than the average of difficult exams reliability [17]. Our results suggest that the differences among students with high scores are low when we use average questions. We suggest that questions should be specifically designed according to the academic level of each group of students. As it is shown in Figs. 3 and 4, the discriminative power of questions with extreme difficulty indices gradually decreases.

Another interesting finding of this study is that MCQ based on clinical vignettes are equally discriminant in students with high or low scores. This finding does not seem to be due to their higher difficulty, since the proportion of questions that were considered as easy was not different between factual and case-based questions. A recent paper found that assessment based on clinical vignettes did not result in greater difficulty or better discriminations among students in a first-year General Pathology course [18]. Other studies have found that clinical vignettes are associated with better discrimination among second year, but not among first-year medical students [19]. This could be the case in our study, that was taken in the third year. The maintenance of the discriminative power by the case-based may be due to the phenomenon of the case specificity. It implies that success on any case is not necessarily transferred to other cases and contexts [20]. The difference between factual and case-based questions is that the first ones explore the ability to recall the correct answer for a question and case-based questions also explore the ability of reasoning in different situations.

It is also likely that part of the difference between both groups of students could be due to common methods variance, that has been defined as the variance that is attributable to the measurement method rather to the construct of interest [21]. In our study, this variance could explain that part of the results obtained by students with a good performance in MCQ tests are related to a special ability to do good MCQ tests.

According to our findings, the lower discriminative power of MCQ exams in groups of students with high scores, could be attenuated increasing the number of case-based questions. Another potential way for correcting this low discriminative power could be to create specific exams for each group, with different difficulty levels, but it is difficult, even for large-scale professional testing organizations, to create exams at a predetermined level of difficulty [17]. Furthermore, exams with different difficulty levels for students in the same course may be unfair. Another potential way of increasing the discrimination capacity of our exams is to reduce the number of distractors. Previous studies have found that most four-option MCQ have at least a poorly functioning distractor [3], and a large meta-analysis concluded that the optimal number of options for MCQ in most settings is three [2]. As it was focused in this study, students with higher performance neglect a large number of distractors. Reducing the number of distractors from 3 to 2 may allow professors to generate better questions with higher discrimination power. This hypothesis should be demonstrated again in further studies.

### Strengths and limitations

To our knowledge, this is the first study showing that the discriminative power of MCQ is lower in groups of students with high scores in medical education. The use of clinical vignettes reduces this loss of discriminative power. Our findings are consistent with other studies that have shown that non-functioning distractors decrease the potential of discrimination, thus, supporting the use of three-option MCQ.

Although we have reviewed many questions that have been used along several years, generalizability of our findings may be limited by several factors. First, the study has been conducted retrospectively in a given School of Medicine with just a few teachers generating the MCQ. Furthermore, the classification of the students in high scorers and low scorers was based on their results in the end-of-semester exam, not in the final exam. Finally, the proportion of questions based on clinical vignettes in our study was low, as compared with the number of factual questions.

## Conclusion

MCQ exams have lower discriminative power in groups of students with high scores. The use of questions based on clinical vignettes may be helpful to maintain the discriminative power of assessment based on MCQ exams.

## Data Availability

The dataset analyzed in the current study are available from the corresponding author on reasonable request.

## Abbreviations

MCQ: Multiple-choice questions
